# Curcumin and Its Carbocyclic Analogs: Structure-Activity in Relation to Antioxidant and Selected Biological Properties

**DOI:** 10.3390/molecules18055389

**Published:** 2013-05-10

**Authors:** Khushwant S. Bhullar, Amitabh Jha, Dani Youssef, H. P. Vasantha Rupasinghe

**Affiliations:** 1 Department of Environmental Sciences, Faculty of Agriculture, Dalhousie University, Truro, Nova Scotia, B2N 5E3, Canada; 2 Department of Chemistry, Acadia University, Wolfville, Nova Scotia, B4P 2R6, Canada; 3 Département des Science, Université Sainte Anne, Church Point, Nova Scotia, B0W 1M0, Canada

**Keywords:** curcumin, antioxidant, tyrosinase, angiotensin converting enzyme, HIV

## Abstract

Curcumin is the major phenolic compound present in turmeric (*Curcuma longa* L.). Curcumin and 15 novel analogs were investigated for their antioxidant and selected biological activities. Strong relationships between the structure and evaluated activity revealed that the compounds with specific functional groups and carbon skeleton had specific biological profiles. Among the compounds tested, the derivatives (*E*)-2-(3,4-dimethoxybenzylidene)-5-((*E*)-3-(3,4-dimethoxyphenyl)acryloyl)cyclopentanone (**3e**), and (*E*)-2-(4-hydroxy-3-methoxybenzylidene)-5-((*E*)-3-(4-hydroxy-3-methoxyphenyl)acryloyl)-cyclopentanone (**3d**) and the parent compound curcumin exhibited the strongest free radical scavenging and antioxidant capacity. Concerning the other biological activities studied the compound (*E*)-2-(4-hydroxy-3-methoxybenzylidene)-5-((*E*)-3-(4-hydroxy-3-methoxy-phenyl)-acryloyl)cyclopentanone (**3d**) was the most potent angiotensin converting enzyme (ACE) inhibitor, while the derivatives (*E*)-2-(4-hydroxybenzylidene)-6-((*E*)-3-(4-hydroxyphenyl)acryloyl)cyclohexanone (**2b**), (*E*)-2-(3,4-dimethoxybenzylidene)-6-((*E*)-3-(3,4-dimethoxyphenyl)acryloyl)cyclohexanone (**2e**) and (*E*)-2-(3,4-dimethoxybenzylidene)-5-((*E*)-3-(3,4-dimethoxyphenyl)acryloyl)cyclopentanone (**3e**) exhibited strong tyrosinase inhibition. Moreover, (*E*)-2-(3,4-dimethoxybenzylidene)-6-((*E*)-3-(3,4-dimethoxyphenyl)-acryloyl)cyclohexanone (**2e**) was also found to be the strongest human HIV-1 protease inhibitor *in vitro* among the tested compounds*.* Cytotoxicity studies using normal human lung cells revealed that the novel curcumin as well as its carbocyclic analogs are not toxic.

## 1. Introduction

**Figure 1:**
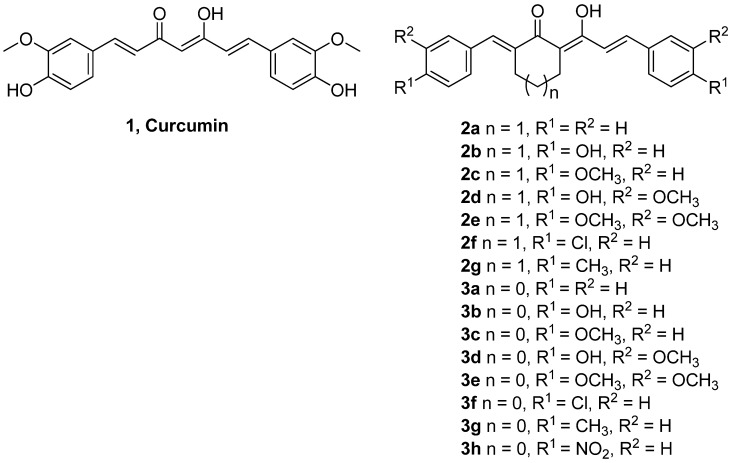
Structures of curcumin and its 15 analogs.

Curcumin [(1*E*,6*E*)-1,7-bis(4-hydroxy-3-methoxyphenyl)-1,6-heptadiene-3,5-dione or diferuloyl-methane, **1**, Figure 1] is a naturally occurring phenolic compound derived from *Curcuma longa* L, commonly called turmeric [1]. Turmeric is an important medicinal ingredient in the Indian system of medicine called Ayurveda and is commonly used as a spice and food preservative. Extensive research during the last few decades has suggested the strong therapeutic and pharmacological potential of curcumin as antioxidant, antimutagenic and antibacterial agent [2]. Curcumin’s strong medicinal properties are also associated with reported anti-cancer [3] and neuroprotective characteristics [4].

Reactive oxygen species (ROS) and free radicals are found to be pathological mediators in many diseases and disorders like diabetes, atherosclerosis, neurodegeneration, rheumatoid arthritis, human immunodeficiency virus (HIV) infection, ischemia and reperfusion injury and obstructive sleep apnea [5,6]. Curcumin exhibits its strong antioxidant activity via its ability to scavenge ROS, produced by catalytic activity of NAD(P)H oxidases and xanthine oxidases along with reactive nitrogen species (RNS) produced by nitric oxide synthase [7]. Apart from its well-studied antioxidant potential, curcumin has also received attention due to its anti-HIV, cardioprotective and other therapeutic properties [8].

Hypertension or high blood pressure is one of the most crucial risk factors for cardiovascular disease (CVD) including myocardial infraction, stroke, renal disease and congestive heart failure. Increased activity of renin angiotensin aldosterone system (RAAS) is involved in high blood pressure. Angiotensin converting enzyme (ACE) is an important enzyme of RAAS and a drug target for treating patients with hypertension [9]. Therefore, compounds with ACE inhibitory activities can be used in treating or preventing hypertension. Studies have concluded that curcumin in combination with ACE inhibitors, extends cardio-protection [10].

Tyrosinase, a copper-containing enzyme is involved in biosynthesis of melanin as it catalyses the *o*-hydroxylation of tyrosine to 3,4-dihydroxyphenylalanine (DOPA) and latter to dopaquinone (*o*-quinone) via oxidation. Melanin pigment is formed subsequently from *o*-quinone by various reactions [11]. Tyrosinase inhibition serves as crucial strategy to treat skin disorders like hyperpigmentation and melanoma. Various tyrosinase inhibitors are used in cosmetics and medicine for the prevention or treatment of pigmentation disorders. Curcumin and its analogs have shown decreased melanin production and tyrosinase inhibition, thus acting as potential anti-melanoma drugs leads [12,13].

HIV is a human pathogen causing AIDS epidemic worldwide with over 40 million people worldwide living with HIV/AIDS [14]. Interestingly, a large number of natural compounds have also been screened for their potential role as antiviral agents for HIV. Highly active anti-retro therapy (HAART) and other drugs have been developed to inhibit enzymes like proteases and reverse transcriptase that facilitate HIV life cycle [15]. Protease enzyme encoded by HIV is essential for viral replication and its inhibition by drugs leads to decrease in number of infectious virus particles [16]. Curcumin and its structural derivatives have been demonstrated to inhibit the HIV viral replication at different stages of its life cycle [15].

The non-toxic food origin and wide range of pharmaceutical properties of curcumin makes it a promising candidate molecule for medicine. Efforts have been made to synthesize new curcumin analogs with stronger antioxidant and biological activities [17,18]. However, the antioxidant activity and biological properties of novel carbocyclic curcumin analogs in relation to structural specifications have not been reported. Our present study aimed at exploring structure-activity relationship for antioxidant, anti-hypertensive, anti-melanoma and anti-HIV properties of curcumin and fifteen novel carbocyclic curcumin derivatives (series **2** and **3**, Figure 1).

## 2. Results and Discussion

The antioxidant activity of curcumin and its fifteen carbocyclic analogs belonging to two series were evaluated by subjecting them to the widely used 2,2-diphenyl-1-picrylhyadrazyl (DPPH^·^) free radical scavenging*,* ferric reducing ability of plasma (FRAP) and oxygen radical absorbance capacity (ORAC) assays*.* The results are presented in Table 1. The anti-hypertensive, anti-melanoma and anti-HIV properties of the compounds under questions were evaluated by subjecting them to angiotensin converting enzyme (ACE), tyrosinase and HIV-I protease inhibitory activity analyses, respectively using standard protocols. These inhibitory potentials of test compounds are presented in Table 2. Finally, to establish the safety profile of the test compounds, their cytotoxicity potential was measured against normal human endothelial lung cells. Appreciable disparity in the chemical/biological activities in these highly related compounds warranted a systematic quantitative structure-activity relationship (QSAR)^REF^ study to understand the effect of structural and electronic properties of the molecules on their antioxidant, anti-hypertensive, anti-melanoma and anti-HIV properties. Physicochemical parameters of the molecule and/or aromatic substituents such as Hammett σ, Hantzsch π, molar refractivity (MR_sub_ for aromatic substituents and MR_mol_ for the whole molecule) and calculated partition coefficients (cLogP), were used for statistical correlation studies (see Section 3.11). The correlation of these physicochemical parameters with the chemical/biological activities was computed by statistical analysis and the results are summarized in Table 3. All of these results are described individually in perspective in the subsequent sections. 

**Table 1:** Antioxidant capacity of curcumin and its structural analogs.

Compound	Antioxidant Capacity *		
	DPPH (IC_50_) (mM)	FRAP (mmole TE/L)	ORAC (mmole TE/L)
**Curcumin**	0.86 ^e^	5.01 ± 0.1 ^c^	1.89 ± 0.2 ^a^
**2a**	2.86 ^h^	1.13 ± 0.4 ^g^	0.12 ± 0.01 ^g^
**2b**	2.58 ^g^	3.02 ± 0.02 ^d^	0.43 ± 0.1 ^f^
**2c**	4.07 ^i^	1.32 ± 0.001 ^f^	0.11 ± 0.01 ^g^
**2d**	0.72 ^d^	5.35 ± 0.1 ^b^	0.50 ± 0.02 ^ef^
**2e**	2.57 ^g^	0.47 ± 0.1 ^k^	0.60 ± 0.1^e^
**2f**	0.71 ^d^	1.28 ± 0.04 ^fg^	0.49 ± 0.1 ^ef^
**2g**	1.36 ^f^	1.12 ± 0.02 ^g^	0.84 ± 0.01 ^d^
**3a**	0.07 ^a^	1.41 ± 0.1 ^f^	1.12 ± 0.1 ^c^
**3b**	0.06 ^a^	2.29 ± 0.1 ^e^	0.49 ± 0.1 ^ef^
**3c**	0.34 ^c^	1.09 ± 0.1 ^gh^	1.17 ± 0.1 ^b^
**3d**	0.07 ^a^	6.70 ± 0.32 ^a^	0.75 ± 0.05 ^d^
**3e**	0.03 ^a^	0.67 ± 0.02 ^j^	0.69 ± 0.1 ^e^
**3f**	0.09 ^a^	1.09 ± 0.1 ^gh^	1.30 ± 0.02 ^b^
**3g**	0.51 ^b^	0.95 ± 0.02 ^hi^	1.25 ± 0.04 ^bc^
**3h**	0.36 ^c^	1.05 ± 0.01 ^ghi^	0.84 ± 0.1 ^d^

* Data are presented as mean ± SD. Mean with different subscripts in each column is significantly different (*p* < 0.05). FRAP, ferric reducing ability of plasma: used 10 μM compounds dissolved in DMSO. ORAC: oxygen radical absorbance capacity: used 1 μM compounds dissolved in DMSO. TE, Trolox equivalence.

**Table 2:** Antihypertensive, anti-tyrosinase, and anti-HIV activities *in vitro* of curcumin and its structural analogs.

Compound	% Enzyme Inhibition ^a^		
	ACE	Tyrosinase	HIV-I protease
**Curcumin**	76.86 ^bc^	12.61 ^k^	48.3 ^c^
**2a**	19.69 ^g^	46.1 ^i^	48.2 ^c^
**2b**	88.73 ^ab^	100 ^a^	55.5 ^b^
**2c**	4.01 ^g^	40.63 ^j^	43.7 ^cd^
**2d**	84.59 ^ab^	56.84 ^h^	37.3 ^ef^
**2e**	36.56 ^f^	100 ^a^	61.6 ^a^
**2f**	ND ^Ω^	60.15 ^ef^	9.9 ^i^
**2g**	15.04 ^g^	59.44 ^fg^	20.1 ^h^
**3a**	51.96 ^ef^	70.85 ^c^	27.0 ^g^
**3b**	90.55 ^ab^	100 ^a^	41.1 ^de^
**3c**	67.42 ^cd^	58.54 ^g^	35.9 ^f^
**3d**	93.89 ^a^	79.99 ^b^	41.8 ^de^
**3e**	56.95 ^de^	100 ^a^	44.5 ^cd^
**3f**	75.12 ^bc^	61.31 ^e^	23.3 ^gh^
**3g**	46.19 ^ef^	67.18 ^d^	22.7 ^gh^
**3h**	88.01 ^ab^	59.22 ^fg^	44.8 ^cd^

^a^ Data are presented as mean. Mean with different subscripts in each column is significantly different (*p* < 0.05). ACE, angiotensin converting enzyme; HIV, human immunodeficiency virus; ^Ω^ ND, not detected; all the enzyme inhibition assays were conducted using 10 μM compounds dissolved in DMSO.

**Table 3:** Correlation constants between antioxidant capacity and extent of ACE, Tyrosinase and HIV-1 protease inhibition displayed by analogs **2** and **3** and certain physicochemical parameters.

Series	Independent	Dependent	Type	Correlation coefficient R	P value
**Series 2**	**Hammett σ**	ACE	Semi-log	−0.827	0.022
		Protease	Semi-log	−0.703	0.078
	**Hantzsch π**	FRAP	Linear	−0.723	0.067
		ACE	Linear	−0.886	0.008
		Protease	Semi-log	−0.750	0.052
	**cLogP**	FRAP	Linear	−0.667	0.101
		ACE	Linear	−0.869	0.011
		Protease	Semi-log	−0.792	0.034
	**Dipole**	ACE	Semi-log	0.687	0.088
	**FRAP**	ACE	Linear	0.795	0.033
**Series 3**	**Hansch π**	FRAP	Semi-log	−0.661	0.074
		ACE	Linear	−0.668	0.070
		Protease	Semi-log	−0.829	0.011
	**cLogP**	FRAP	Linear	−0.614	0.105
		ORAC	Linear	0.882	0.004
		Protease	Semi-log	−0.874	0.005
	**Dipole**	DPPH	Semi-log	−0.622	0.099
		ORAC	Linear	−0.620	0.101
	**DPPH**	Tyrosinase	Semi-log	−0.627	0.096
	**FRAP**	ACE	Linear	0.591	0.123

### 2.1. DPPH^·^ Scavenging Assay 

DPPH**^·^** assay is widely used to observe radical scavenging activity of various compounds and extracts. DPPH**^·^** is stable N radical which is reduced to hydrazine during its reaction with hydrogen donors. Several reports in literature highlight the DPPH**^·^** scavenging activity of curcumin, its analogues and amino acid conjugates [2,7]. In the present study, strong DPPH**·** scavenging activity was observed in all of the curcumin derived compounds (Table 1). The IC_50_ values were determined using linear trends emerging from DPPH**^·^** scavenging ability of curcumin derivatives at different concentrations. Among curcumin analogues tested, all series **3** compounds showed potent DPPH**^·^** scavenging activity surpassing Curcumin, representing IC_50_ values in the range from 0.03–4.07 mM of compounds. Compound **3e** with anisol substitution at R1 and R2 exhibited strongest free radical inhibition (IC_50_ = 0.03 mM) while compound **2c** showed the lowest free radical inhibition (IC_50_ = 4.07 mM) (*p* < 0.05). Several of the series **3** compounds, *viz.* **3e**, **3b**, **3a**, **3d** and **3f** (in that order) showed over 10-fold higher radical scavenging capacity than curcumin in the *in vitro* assay. The presence of constrained cyclopentane ring in curcumin derivatives led to high antiradical capacity. Also, the presence of hydroxyl and methyl ether functionalities on the aromatic rings appeared to impart the antiradical capacity to these compounds. Although superior to curcumin, compounds **3c** with 4-methoxy, **3g** with 4-methyl and **3h** with 4-nitro groups showed approximately 10-fold lower antiradical capacity than other of curcumin analogues in series **3**. These results are in accordance with a previous study [19] which reported strong radical scavenging activity of curcumin along with its structural analogues. The two series **2** cyclohexanes analogs which showed comparable antiradical activity to curcumin are **2d** (R_1_=OH, R_2_=OCH_3_) and **2f** (R_1_=Cl, R_2_=H). Rest of the series **2** compounds were found to be inferior to curcumin with **2c** (R_1_=H, R_2_=OCH_3_) displaying ~5-fold weaker DPPH**^·^** scavenging capacity. 

The QSAR study using statistical analysis looking at the effect of physicochemical constants (Hammett σ, Hantzschπ, MRsub, MRmol, cLogP, dipole, summarized in Table 3) on the DPPH**^·^** scavenging activity did not result in any meaningful correlation for series **2** molecules. For series **3** molecules, however, an inverse trend towards significance (R value −0.622, P value 0.099) was noted for correlation between DPPH**^·^** scavenging activity and molecular dipole. This means higher the molecular dipole, lower is the DPPH**^·^** scavenging IC_50_. This confirms that polar molecules with multiple hydroxyl groups showed potent radical scavenging capacity but the overall molecular shape also appear to play a role as series **2** compounds with similar substituents were not found to be equally active. The molecular shapes of series **3** compounds are expected to be somewhat different from those of series **2** compounds. This was demonstrated using molecular modeling in our previous report [3].

### 2.2. Ferric Reducing Ability of Plasma (FRAP) Assay

The FRAP assay uses antioxidants in a redox-linked colorimetric assay to reduce ferric tripyridyl triazine to ferrous form at low pH [20]. Use of FRAP assay to decipher the antioxidant potential of curcumin and its phenolic analogues has been reported previously [21]. In the present investigation, FRAP assays were conducted on curcumin and its structural analogues at 10 µM concentration. The results are presented in Table 1. Three compounds, namely curcumin (**1**), **3d** and **2d**, exhibited the highest antioxidant capacity at 10 µM concentration as observed from the FRAP values ranging from 0.47–6.47 mM TE/L. However, compound **2e** (R_1_=OCH_3_, R_2_=OCH_3_) showed the lowest antioxidant capacity (0.47 mM TE/L) among all analogues tested (*p* < 0.05). Unlike DPPH**^·^** scavenging activity, only two compounds **3d** (R_1_=OH, R_2_=OCH_3_) and **2d** (R_1_=OH, R_2_=OCH_3_) surpassed curcumin as potent antioxidants. It was observed that presence of 3-OCH_3_ and 4-OH substituents (as in curcumin) plays a vital role in imparting higher antioxidant capacity in compounds. Analogues with mono-substituted aromatic rings, even with hydroxyl or methoxy groups, followed a decreasing trend in terms of FRAP values. Similar to DPPH**^·^** scavenging ability, curcumin analogues with constrained cyclopentane ring exhibited stronger antioxidant capacities. Analogues with electron withdrawing chloro or nitro groups showed very low antioxidant capacity. Our QSAR study indicated that the FRAP values for test compounds showed negative linear correlation with Hansch π and cLogP for both series of compounds (Table 3). This signifies that higher the lipophilicity, lower the FRAP values or the antioxidant activity. Phenolic compounds are expected to have lower lipophilicity and therefore according to this correlation, have shown higher antioxidant capacity.

### 2.3. Oxygen Radical Absorbance Capacity (ORAC) Assay

The performance of curcumin and its analogues in the ORAC test in the present study is presented in Table 1. Curcumin had the strongest peroxyl radical scavenging capacity (5.01 mM TE/L) when compared to other curcumin analogues. The results showed that curcumin followed by compounds **3c** and **3f** showed strongest antioxidant capacity (1.09 mM TE/L). The series **2** compounds, in general, showed poor ORAC values (0.11–0.84 mM TE/L). Consistent with its lowest antiradical capacity (DPPH assay), **2c** exhibited the weakest antioxidant capacity (*p* < 0.05). In comparison of antioxidant capacities of curcumin derivatives, series **3** analogues with cyclopentane ring including **3f**, **3g** and **3c** showed comparatively stronger antioxidant capacities when compared to other analogs tested. The presence of -OH or -OCH_3_ on benzene rings of series **3** compounds contributed to high antioxidant capacities of these compounds. Compound **3e** (R_1_=OCH_3_, R_2_=OCH_3_) had weaker ORAC values as compared to curcumin and other series **3** compounds and was found to be consistent with its FRAP values. In the QSAR studies (Table 3), the ORAC values were found to correlate with cLogP and dipole of series **3** compounds only. Positive linear and significant correlation with cLogP indicates that high lipophilicity leads to high ORAC values of series **3** compounds and this is further substantiated by negative linear correlation with dipole which is inversely related to cLogP. Results were similar to earlier studies [20] who also observed strong antioxidant capacity of curcumin. Recent reports [22,23] have also demonstrated that curcumin and its structural analogues as strong free radical scavengers.

### 2.4. Angiotensin Converting Enzyme (ACE) Inhibition Assay

ACE is a membrane bound protein which plays a crucial role in the regulation of renin-angiotensin system in relation to maintaining normal blood pressure and electrolyte balance [9] ACE has dual function, a vasoconstrictor by conversion of angiotensin I to angiotensin II, as well as a vasodilator by inactivation of bradykinin. This phenomenon leads to regulation in arterial blood pressure. Previous studies [10,24] have also described strong cardio-protective properties of curcumin. Thus, it is logical to test curcumin analogs for their ACE inhibition potency which may be the mode of cardio-protection by curcumin. In the present study, it was observed that curcumin and most of its analogues belonging to both series had strong ability to inhibit ACE (Table 2) at the concentration of 10 µM. Compound **3d** (R_1_=OH, R_2_=OCH_3_), which also had strong antioxidant capacity, exhibited the greatest ACE inhibition (93.89%) among all 16 compounds studied (*p* < 0.05). Among other compounds with notable ACE inhibition activity were **3b**, **2b**, **2d** and **3h**, each exhibiting superior activity than Curcumin (76.86%). No ACE inhibition activity was detected for compound **2f** (R_1_=Cl, R_2_=H) followed by negligible activity in compound **2c** (4.01%) under test conditions. 

All of the series **3** compounds displayed appreciable ACE inhibition with polar hydroxylated compounds leading the trend. Our QSAR study (Table 3) revealed a number of meaningful correlations between ACE inhibition values and physicochemical properties including their measured FRAP values. The ACE inhibition activities of both series of compounds correlated negatively with Hansch π. This implies that the lipophilic compounds show lower ACE inhibition. This corroborates with the observation that relatively hydrophilic –OH substituted compounds showed higher ACE inhibition capacity. FRAP values, which are a measure of antioxidant capacity, showed linear and positive correlation with ACE inhibition potency for both series of compounds signifying that higher antioxidant activity imparts stronger ACE inhibition ability to the compounds. Hammett σ values for series **2** compounds correlated negatively with ACE inhibition. This means that electron withdrawing substituents (such as Cl, higher Hammett σ value) on the aromatic ring are detrimental to the ACE inhibition capacity. Negative correlation was also shown by cLogP for series **2** compounds with ACE inhibition implying that as lipophilicity decreased, the superior ACE inhibition capacity was displayed by the test compounds. The exact same inference can be drawn from the positive correlation between ACE inhibition and dipole of series **2** compounds. All these data suggest that ACE inhibition capacity of curcumin analogs can be enhanced by (a) placing electron releasing groups on aromatic ring (low Hammett σ), (b) making the molecule less lipophilic (high Hantzsch π and cLogP) and more polar (high dipole), and (c) by improving the antioxidant activity (high FRAP values). This provides an excellent insight into the design of potent ACE inhibitors in future based on curcumin framework.

### 2.5. Tyrosinase Inhibition Assay

Tyrosinase is an important enzyme involved in melanin synthesis and human melanoma. Tyrosinase is involved in catalytic conversion of tyrosine to DOPA and oxidation of DOPA to DOPA quinone [11,13]. Since the conversion involves a redox process, curcumin analogs as potential antioxidants are logical candidates for testing their tyrosinase inhibition activity. Earlier studies [12,13] have revealed that curcumin and its analogs are potent melanogenesis inhibitors. 

Interestingly, it was observed that all curcumin analogues exhibited higher tyrosinase inhibition activity than curcumin at the concentration of 10 µM (Table 1). Compounds such as **2b**, **2e**, **3b** and **3e** exhibited highest and complete inhibition of tyrosinase at 10 µM concentration. However, curcumin showed lowest inhibition of tyrosinase (12.61%) among all compounds (*p* < 0.05). Similar to antioxidant potential, series **3** compound showed the stronger inhibition of tyrosinase *in vitro*. The tyrosinase inhibition studies confirmed the biological application of the novel compounds as all compounds inhibited the enzyme activity *in vitro*. The common feature that the four complete inhibitors of tyrosinase (**2b**, **2e**, **3b** and **3e**) is a hydroxyl substituent at position 4 of the aromatic rings. These curcumin analogues warrant further evaluation for prevention/treatment of human melanoma, skin whitening products, and food preservatives. The statistical analysis resulted in only one meaningful correlation of tyrosinase inhibition potency and its negative correlation with DPPH values (Table 3). This meant that compounds with high radical scavenging activity (low DPPH IC_50_) showed high tyrosinase inhibition, which was expected.

### 2.6. HIV-I Protease Inhibition

HIV is a global pandemic and has led to loss of millions of lives worldwide [14]. The enzymes like HIV integrase and protease hold key importance in virology as modern research aims in developing clinically active viral enzyme inhibitors in order to terminate viral life cycle [15,16]. Previous studies [15,25] have shown antiviral and protease inhibitory properties of curcumin and its structural derivatives. In this investigation, HIV protease inhibition was found to be the highest in **2e** (61.6%) followed by **2b** (55.5%). Rest of the compounds showed nearly equivalent (**2a**, **2c**, **3e** and **3h**) or inferior protease inhibitory activity than curcumin (Table 2). Like other properties investigated, the presence of -OH or -OCH_3_ substituents on the aromatic rings did appear to impact higher anti-HIV-1 protease activity to the compounds. However, contrary to other activities reported in this paper, series **3** compounds were shown to be inferior to series **2** compounds (*p* < 0.05). This may also be a manifestation of the divergence in the overall molecular shapes in the two series of compounds [3].

The HIV-I protease inhibition assay also showed potency of novel curcumin analogs in inhibiting the viral life cycle enzyme. With regards to the QSAR studies (Table 3), it was noted that the HIV-1 protease inhibitory activity of both series of compounds correlated negatively with Hantzsch π and cLogP values. This is consistent with the observation that less lipophilic compounds have shown higher HIV-1 protease activity. Also, for series **2** compounds, Hammett σ values for the aryl substituents showed negative correlation with the anti-HIV-1 protease activity. This again suggests that electron donating substituents (e.g., -OH or -OCH_3_) imparted higher protease inhibition properties to these molecules. 

### 2.7. Cytotoxicity Analysis

**Figure 2:**
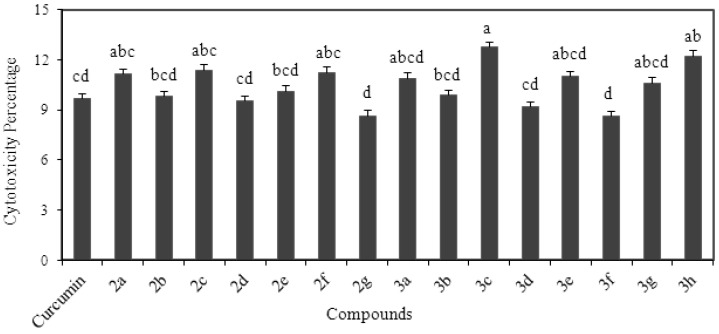
Cytotoxicity analysis of curcumin and its analogs using human endothelial lung cells.

In the current study authors conducted a toxicology study to confirm safety of compounds and their dosage for potential animal or clinical studies. The study was conducted at 10 µM concentration using human endothelial lung cells. Toxicity of the compounds was verified by LDH release assay and results are shown in Figure 2. All the compounds showed very low cytotoxic manifestations in human endothelial lung cells. The cytotoxic membrane damage by curcumin analogs on human endothelial lung cells ranged between 8.6 to 12.7 %. The membrane LDH release assay showed that the highest cytotoxic behavior was shown by compound **3c** (R_1_=OCH_3_, R_2_=H) while compounds **2g** (R_1_=CH_3_, R_2_=H) and **3f** (R_1_=OCH_3_, R_2_=OCH_3_) exhibited the lowest cytotoxicity effects (*p* < 0.05). However, other compounds including curcumin exhibited very low toxic behavior but were significantly different among each other (*p* < 0.05). The cell study indicated that both series **2** and **3** compounds had no toxic effects in the normal human cells. Literature reports on cytotoxic behavior of potent natural antioxidants are rare. Research in the field of free radicals mainly deals with screening of novel antioxidants and their mechanism of action. The study showed that all compounds exhibited very low level of the cytotoxicity. The compounds of both series driven from curcumin appeared as nontoxic drug candidates. 

## 3. Experimental

### 3.1. Materials

Curcumin (65–70%) was purchased from Sigma Aldrich Canada Ltd. (Oakville, ON, Canada) and was further purified by column chromatography to achieve >95% purity (as measured by ^1^H-NMR). The re-syntheses and purification of all curcumin analogues (**2a**–**g**, **3a**–**h**) used in this investigation was performed according to earlier report of our lab [18]. Ferric chloride, 2, 2-diphenyl-1-picrylhyadrazyl, Trolox, 2,2'-azobis (2-amidinopropane) dihydrochloride (AAPH), DMSO, fluorescein sodium salt, ferric tripyridyl triazine (Fe III TPTZ), sodium carbonate and enzymes including ACE, tyrosinase and HIV-1 Protease were also purchased from Sigma Aldrich. Promega Cytotox 96® non-radioactive colorimetric assay kit was purchased from Promega Corporation (Madison, WI, USA). Dulbecco’s Modified Eagle’s Medium (DMEM), penicillium, streptomycin and cell culture tested DMSO was purchased from American Type Culture Collection (Manassas, VA, USA).

### 3.2. Sample Preparation

All compounds were dissolved in DMSO (Sigma Aldrich) and then sonicated for one minute to attain adequate homogeneity in reaction mixtures. Stock solutions of compounds (1 mM) were stored in amber vials for further analysis. DMSO was used for making working solutions of various concentrations. Analysis of curcumin and its analogs for selected biological properties and toxicity analysis was conducted at 10 µM concentration as our previous work [3] with these compounds showed cytostatic activity at ~10 µM concentrations. In order to further understand biological properties of these compounds and confirm their non-toxic manifestations, analyses were performed at 10 µM concentration.

### 3.3. Cell Culture

Human lung cell line (ATCC^®^ PCS-100-022^™^) was purchased from American Type Culture Collection (Cederlane, Burlington, ON, Canada). Cell lines were maintained and grown in DMEM complete growth media, supplemented with 10% fetal bovine serum (FBS), 100 units/mL penicillin and 100 µg/mL streptomycin (Cederlane) at 37 °C, in a humidified 5% CO_2_ incubator. Cell growth medium was changed every 48 hours and when cells reached 80% confluence, they were detached from 75 cm^2^ tissue culture flask using 0.25% trypsin-EDTA treatment. The detached cells were collected in sterile tube in FBS free media. Collected cells were then counted using the Millipore Scepter 2.0 handheld automated cell counter. Further assay specific analysis was performed as described in section 2.10.

### 3.4. DPPH^·^ Scavenging Assay

Antiradical activities of curcumin and its various structural analogues were determined using the free radical 2, 2-diphenyl-1-picrylhyadrazyl (DPPH**^·^**) scavenging assay, which was performed using a previously described method [26] with slight modifications. Initially, 0.2 mM DPPH**^·^** solution was prepared using DMSO as the solvent. To initiate the reaction, 150 µL of 0.2 mM DPPH**^·^** solution and 150 µL curcumin derivatives at different concentrations were pipetted into a 96-well clear polystyrene plate (Costar 3395, Fisher Scientific, Ottawa, ON, Canada). During reduction of DPPH**^·^** solution by antiradical compound, absorption of DPPH**^·^** was measured at 515 nm. The spectrophotometric data was acquired using BMG FLUOstar OPTIMA spectrophotometer (BMG Labtech, Durham, NC, USA) and the IC_50_ values were calculated.

### 3.5. Ferric Reducing Ability of Plasma (FRAP) Assay

FRAP assay is based on ferric to ferrous ion reduction to test the electron donating capacity of antioxidants [19]. The assay was modified by using the 96-well microplate reader with an injection port system [27]. Briefly, the FRAP working reagent was made using acetate buffer, 2,4,6-Tris (2-pyridyl)-s-triazine solution and ferric chloride in the ratio 10:1:1. Working reagent was injected into the wells containing curcumin analogues using an injector port of the plate reader (FLUOstar OPTIMA plate reader, BMG Labtech). After 6 min reaction time, absorbance was measured at 593 nm. Trolox standards at different concentrations were used to calculate the antioxidant capacity as mmol Trolox equivalence per L.

### 3.6. Oxygen Radical Absorbance Capacity (ORAC) Assay

ORAC assay is based on the free radical damage to a fluorescence probe measured by fluorescence intensity over the time [20]. The fluorescein sodium salt was used as fluorescence probe and change in its intensity was estimated as the index of the degree of free radical damage. Assay was performed using method described elsewhere [28]. Briefly, the standards and samples were prepared using 75 mM phosphate buffer (pH 7). As the peroxy radicals are generated from decomposition of 2,2'-azobis(2-amidino-propane) dihydrochloride (AAPH), the signal was quenched from fluorescein. However, in the presence of antioxidant samples the fluorescence signal can be stabilize which depends directly upon the antioxidant’s capacity to neutralize the free radical. Quadratic relation emerging from florescent area under the decaying curve was used to calculate antioxidant capacity as mmol Trolox equivalence per L. 

### 3.7. Angiotensin Converting Enzyme (ACE) Inhibition Assay 

ACE assay is based on ACE inhibitory activity of test samples. The enzyme activity was measured by production of hippuric acid from the substrate hippuryl-L-histidyl-L-leucine (HHL) [9]. ACE cleaves the substrate to expose a free N-terminus, which was fluorogenically labeled with *o*-phthaldialdehyde (OPA). The florescence signal was measured by using excitation of 350 nm and emission of 500 nm. ACE inhibition activity of samples was measured using the following equation:

% inhibition = 100 − (**A**_control_ − **A**_sample_)/**A**_control_ × 100 (1)

where **A** is the absorbance Equation (1).

### 3.8. Tyrosinase Inhibition Assay

Tyrosinase inhibition was calculated based on previously described methods using l-DOPA as the substrate [29]. Tyrosinase inhibition depended upon the amount of dopachrome produced in the reaction mixture. Absorption was measured at 490 nm to calculate the amount of dopachrome produced. The assay was performed in 96-well clear polystyrene plates using FLUOstar OPTIMA plate reader. The tyrosinase inhibitory effect of sample was measured using the following the Equation (1). 

### 3.9. HIV I Protease Inhibition Assay

HIV-1 protease inhibition was performed by using a previously described method [30]. HIV protease fluorescent substrate and HIV-1 protease enzyme (Sigma Aldrich) were used to determine HIV inhibition capacity of sample. Three primary controls *i.e.*, blank, sample and positive control were used. Reaction mixture of 200 µL comprised of five enzyme units (5U) of HIV-1 protease along with 0.5 pmol of enzyme substrate. After addition of both enzymes and substrates, test compounds were added in different concentrations. Reaction mixture was incubated at 37 °C which leads to generation of fluorescence signal by cleaving Tyr-Pro peptide bond of the substrate. Finally reaction mixture was heated for 5 minutes at 90 °C to stop the reaction. The mixture was centrifuged at 12,000 rpm for 3 min. and supernatant was used to measure the fluorescence signal. The enzyme inhibition was calculated using the Equation (1).

### 3.10. Cytotoxicity Analysis

Cytotoxicity assay was performed using Promega Cytotox 96^®^ non-radioactive colorimetric assay (Promega Corporation). The assay measures the lactate dehydrogenase (LDH) release upon cell lysis. The released LDH from cells was measured in an enzymatic assay based on conversion of tetrazolium salt to a red formazan product. Briefly, 5,000 human endothelial lung cells in 100 µL of serum-free culture medium were seeded in 96 well sterile plates. Cells were allowed to adhere to the plates for 24 h at 37 °C in a humidified, 5% CO_2_ incubator. After 24 h, the serum-free media was replaced by test compounds in 100 µL of serum-free culture medium. An assay control and a positive control containing human endothelial lung cells were setup according to the manufacturer’s instructions. The test compounds at 10 µM concentration were allowed to interact with cells for 24 hours after which absorbance were recorded at 490 nm using BMG FLUOstar OPTIMA spectrophotometer (BMG Labtech, Durham, NC). The results were obtained using the following equation:

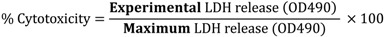
(2)


### 3.11. Quantitative Structure Activity Relationship Studies Using Statistical Analysis

In order to verify whether the antioxidant capacity and the enzyme inhibition studies conducted correlated with one or more physicochemical properties of the aryl substituents [31] and/or the whole molecule in both series of compounds, linear and semi-logarithmic correlations were obtained (Minitab® from Minitab Inc., State College, PA, USA) between Hammett σ values, the Hansch π values and molar refractivity (MR_sub_) constants (reflect the electronic, hydrophobic and steric properties of the aryl substituents, respectively). Three other calculated (Chem3D^®^ from CambridgeSoft Corporation, Morrisville, NC, USA) parameters for the whole molecule, viz. cLogP (octanol/water partition coefficient) and dipole (polarity) and molar refractivity (MR_mol_) were also correlated with antioxidant activity and extent of enzyme inhibition. The antioxidant activity data was also correlated with the extent of enzyme inhibition data. Only the significant correlations obtained for both the series of molecules. The value of P in the range 0.05–0.1 indicates a trend towards significance, while values < 0.05 suggest significant correlation. The magnitude of coefficient r shows the extent (the closer the values to 1, the better) and the nature (sign positive or negative) of the correlation.

### 3.12. Statistical Analysis

Analysis was carried out using a complete randomized design and treatments were run in triplicates (n = 3). Statistical analysis was done using analysis of variance (ANOVA) method of general linear model (GLM) procedure using SAS V8 (Cary, NC, USA). Normal distribution and constant variance was checked and Tukey’s studentized range at α = 0.05 was used to determine significant differences.

## 4. Conclusions

Curcumin and fifteen constrained curcumin structural analogs with various functional groups attached to their aromatic rings were analyzed for their antioxidant activity and selected biological properties relevant to antioxidant activity *in vitro*. These studies indicated that curcumin and its novel analogs are strong antioxidants and free radical scavengers. Compound **3e**, had the strongest antiradical capacity (DPPH**^·^** assay) while **3d** and curcumin exhibited strongest antioxidant capacity (FRAP assay). Compounds such as **2d** and **3d** along with curcumin were shown to possess potential to control and neutralize oxidative stress (FRAP assay). Compound **3d** exhibited strongest *in vitro* inhibition of ACE activity suggesting its potential in treating hypertension. Studies on *in vitro* tyrosinase inhibition showed **2b**, **2e**, **3b** and **3e** having strongest potency. Interestingly, **2e** also exhibited the strongest *in vitro* HIV-1 protease inhibition properties. As expected, the presence of phenolic and methoxy groups imparted desirable antioxidant and other biological properties to the tested compounds. This was further corroborated by the QSAR studies which clearly indicated that electron donating polar substituents improved the potency of the test compounds in all the properties examined. It also provided further insight into designing compounds with structural features that may translate into higher activity. Toxicology studies using human endothelial cells confirmed the non-toxic manifestations of curcumin and its novel analogs. Our studies have established that these nontoxic curcumin analogs, with appropriate structural modifications, have the potential to be developed as vasodilators, anti-melanoma and antiviral drugs and further investigations in this direction are warranted.
